# Efficacy of Topical Enalapril in Treatment of Hypertrophic Scars

**DOI:** 10.29252/wjps.7.3.326

**Published:** 2018-09

**Authors:** Ali Akbar Mohammadi, Ali Parand, Sina Kardeh, Mansour Janati, Soheil Mohammadi

**Affiliations:** 1 Burn and Wound Healing Research Center, Division of Plastic and Reconstructive Surgery, Department of Surgery, Shiraz University of Medical Sciences, Shiraz, Iran;; 2 Student Research Committee, Shiraz University of Medical Sciences, Shiraz, Iran;; 3 Cell and Molecular Medicine Student Research Group, Shiraz School of Medicine, Shiraz, Iran;; 4 Department of Cardiac Surgery, Shiraz University of Medical Sciences, Shiraz, Iran;; 5 Faculty of Medicine,Tehran University of Medical Sciences, Tehran, Iran

**Keywords:** Hypertrophic scar, Enalapril, Burn

## Abstract

**BACKGROUND:**

Angiotensin II activation by angiotensin-converting enzyme (ACE) is a significant mediator in wound healing and collagen production. In this study, the effect of topical application of ACE on hypertrophic scar formation has been studied in a clinical trial.

**METHODS:**

Thirty patients with hypertrophic scar and itching after treatment of 2^nd^ or 3^rd^ degree burns participated in this double-blinded clinical trial. Subjects had two same-degree scars on symmetrical sites of body which were randomly allocated into two groups. One side was treated with 1% enalapril ointment and the other side with placebo twice daily. During a 6-months follow-up, a scoring table for itching was completed on a daily basis by patients. Furthermore, a single surgeon measured size of scars once a month. The mean size, thickness and itching score were calculated for each scar and compared between medication and placebo-treated scars.

**RESULTS:**

The mean size of scars in enalapril treated side was significantly less than scars in the placebo side. Additionally, enalapril treated scars had significantly lower itching scores compared to the placebo group.

**CONCLUSION:**

Topical enalapril significantly decreases the clinical parameters of hypertrophic scar and also itching as an indirect indicative of scar improvement. Furthermore, enalapril proved to be clinically safe for patients with low incidence of adverse drug reactions and acceptable cost effectiveness.

## INTRODUCTION

Hypertrophic scaring (HTS) is a major clinical problem in both developing and industrialized worlds with manifestations of a dysfunctional form of dermal fibroproliferative disorders caused by exuberant wound healing response to skin injuries including burn, abrasions, laceration, surgery and trauma.^1^ The main characteristic of HTS is disruption of the exquisite balance of degradation and deposition of fibroblast-derived extracellular matrix (ECM) proteins, mainly by excessive collagen production.^2^ Based on the expression of α-SMA, fibroblasts phenotype in HTS differs from the normal ones. These cells may also differentiate into myofibroblasts which are more potent to generate collagen, less sensitive to apoptotic signals and thus play a key role in HTS formation.^3^

Besides, increased vascularization and emergence of prominent vertically oriented blood vessels, replacement of the papillary and reticular dermis by scar tissue, flattening of the epidermis, hypercellularity and reduction of small leucine-rich proteoglycans (SLRPs) are frequent features.^4^ HTS occurs as a result of prolonged inflammatory response to injury and persistent fibrosis which results in the pathological characteristics of hypertrophic scars including a red, elevated and rigid scar associated with cutaneous and joint contractures, pruritus, and prolonged pain. In addition to these complications, the stigma of cosmetic disfigurement is another important concern for patients and surgeons which may lead to psychological and social issues.^5^^,^^6^

Although HTS involves the deep dermis, there are no expansions into surrounding tissues and beyond the general margins of the wound. Further, the initial site of the injury may also undergo limited spontaneous resolution over time.^7^ The incidence of HTS following different types of injuries is not known; however, it is a frequent outcome that creates obstacles of immense magnitude. Considering that hypertrophic scars are common complication of burn injury, approximately four million patients suffer scar formation due to burns in the developed world each year, while the incidence is even greater in developing countries.^8^

HTS has equal sex distribution with the highest occurrence in the second and third decades. Unlike patients suffering from keloids, genetic predisposition has not been suggested for hypertrophic scars, as reports of positive family history are rare. Previously, diverging incidences have been reported for HTS, with rates from 40% to 70% after surgical procedures and up to 90% following burn injuries, depending on the depth of tissue damage.^9^^,^^10^ Despite the introduction of a wide variety of management protocols for hypertrophic scars, no definitive treatment has been established with optimal clinical results so far. Hence, patients may need combination therapeutic modalities based on size, depth and location of the lesion and response to therapy.^11^

Accepted treatment options for HTS include surgical excision of the scar with or without grafting, topical and intralesional corticosteroids injections, interferon therapy, bleomycin, silicone gel sheeting, laser therapy, compression therapy and over-the-counter treatments such as onion extract.^12^ Although among these methods intralesional steroid therapy is regarded as the benchmark treatment, complications with steroid injections such as repetitive painful injections and unpredictable efficacy are widely reported.^13^

Thus, further evaluation for prospective treatments that can be simply applied while achieving acceptable efficacy seems essential. ACE inhibitors hinder the conversion of angiotensin I to its active form, angiotensin II. Previously, many studies exhibited the angiotensin II dependent fibroblast proliferation, matrix metalloproteinase activity suppression and also collagen production in cardiac tissue especially left ventricular collagen content in heart failure.^14^^-^^16^ Moreover, it has been noted that inhibition of angiotensin II can diminish pathologic fibrosis in many organs including heart, kidney and lung.^17^^-19^

Further, recent investigations have demonstrated local overactivity of ACE and angiotensin II in wound healing and skin fibrosis. This can be evidenced by higher levels of ACE and angiotensin II in hypertrophic scars compared to normal scars and normal skin.^20^ Currently, medical evidence on the potential of ACE inhibitors as clinical choices for treating hypertrophic scars is limited to animal studies and case reports.^21^ Since there exists a gap between the theoretical basic science research and the limited clinical studies to introduce ACE inhibitors as effective therapeutic options, in this study we decided to evaluate the efficacy of enalapril, a potent ACE inhibitor, in reduction of hypertrophic scar size and itching as common complications in burn patients.

## MATERIALS AND METHODS

In this single-center, prospective clinical trial study, conducted at the Shiraz Burn and Wound Healing Research Center, Division of Plastic and Reconstructive Surgery, Department of Surgery, Burn Hospital, Shiraz, Iran, 30 patients who developed hypertrophic scar and itching after treatment of 2^nd^ or 3^rd^ degree burns have participated. Subjects were selected among patients who had two same-degree scars on symmetrical anatomic sites of body, so that medication and placebo could be administered simultaneously on either side for each patient. 

Exclusion criteria consisted of incomplete wound healing, cardiovascular diseases, renal failure and pregnancy. Patients were required to fill out an informed written consent prior to participation in the study. All processes were approved by Ethics Committee of Shiraz University of Medical Sciences. Information were obtained via questionnaires inquiring about gender, age, cause of burn, site of scars, the time interval between wound healing and beginning of the experiment and duration of medical therapy. Scars were randomly allocated into two groups. One group was treated with enalapril, while the other one received placebo. Thus, each patient was also considered as his or her control. 

To obtain 1% ointments, 1 gr powder of enalapril was dissolved in 100 gr of basic ointment (Eucerin). Eucerin was used as placebo. In order to double-blind the study, the ointment and placebo were transferred into unlabeled containers with different colors. Hence, neither the surgeon nor patients could distinguish them apart. Patients were educated to evenly apply equal volumes of ointment from containers; each only on the scar of one side and twice daily for a period of 6 months. 

A scoring table for itching (from 0=no itching, to 4=severe itching) was completed on a daily basis by patients. Furthermore, a single surgeon measured size and thickness of scars during monthly examinations. The mean thickness and itching scores were calculated for each scar and compared between medication and placebo-treated scars. Statistical analysis was done by SPSS software (Version 20, Chicago, IL, USA) using the Mann–Whitney U test. Statistical values *p*<0.05 were considered significant.

## RESULTS

Among these 30 patients, female and male patients both constituted 50% (N=15) of cases. Differences in age (*p*=0.71), the time interval between wound healing (*p*=0.35) and initiation of the experiment and duration of medical therapy (*p*=0.32) were not significant. Flame burn was responsible for 24 cases (80%), whereas 6 patients (20%) had mentioned scald burn. The distribution of the sites of burn was 5 (16.7%) in arm, 12 (40%) in forearm, 2 (6.7%) in trunk, 7 (23.3%) in thigh, and 41 cases (13.3%) in the leg.

There were no significant differences in the mean scar size between the enalapril and placebo groups at the beginning of the study (*p*>0.05). In the final follow-up assessment, enalapril-treated scars (2.02±0.55) were significantly smaller in size compared to placebo side (2.30±0.64) (*p*<0.001). Initial evaluation of itching scores between the enalapril and placebo groups did not indicate any significant differences (*p*>0.05). The mean itching score proved to be lower in enalapril-treated side (1.73±0.69) compared to the placebo side (2.43±0.67) after completion of treatment (*p*<0.001).

## DISCUSSION

As an important problem in clinical practice and a disfiguring form of wound healing with serious functional and psychological complications, many advances have been made in the understanding of hypertrophic scar formation and development of several pharmacological and nonpharmacological methods of treatment. However, the drawbacks such as high cost (silicone gel sheeting, bleomycin and interferon), and recurrence (surgery, and 5-fluorouracil) are are still a major impediment to increased adoption of these methods. Besides, the precise mechanism of hypertrophic scar is yet to be elucidated in the cellular level and molecular level.^22^

The pathogenesis of hypertrophic scars includes excessive and abnormal composition and metabolism of collagen and other extracellular matrix components in the wound site. The transforming growth factor (TGF)-β is a family of cytokines involved in various essential cellular functions including wound healing biology and the pathophysiology of hypertrophic scarring. Although TGF-β has three isoforms (TGF-β1, -β2, -β3) as as inactive latent precursors that might appear to have overlapping signaling pathways in vitro, their different level of expression in vivo leads to different roles in skin homeostasis. The main processes that are regulated by TGF-β in wound healing are angiogenesis, inflammation, fibroblast proliferation, collagen production and deposition and remodeling of the ECM.^22^

In the inflammatory phase platelets release different growth factors and cytokines, such as epidermal growth factor (EGF), fibroblast growth factor (FGF), platelet-derived growth factor (PDGF), and TGF-β1 which function as chemoattractants or promoters of the inflammatory cascade. These factors along side complement particles and serum factors also mediate leukocyte migration and their transformation to the monocytes in the wounded area. Furthermore, while macrophages clear the area of debris they also release crucial cytokines including VEGF which in turn initiate the formation of granulation tissue and angiogenesis.^23^

PDGF and TGF-β1 are 2 main role players in the production of key ECM components, such as collagens and fibronectin. On the other hand, macrophages and neutrophils cause collagen breakdown by releasing matrix metalloproteinases (MMPs). However, TGF-β1 inhibits MMP synthesis and cause greater accumulation of collagen fibers.^24^ Angiotensin II can cause significant increases in expression of TGF-β1 which is an important mediator of the cardiac hypertrophic growth response. Schultz *et al.* showed impaired cardiac function and also more than 20% increase in left ventricular mass of angiotensin II –treated wild-type mice.^25^

ACE, angiotensin II and angiotensin type 1 receptors all have increased expressions in human hypertrophic scar fibroblasts.^26^ Morihara *et al.* sought to investigate ACE activity in normal human skin, wounded skin, and pathologic scars. The results showed that the ACE activity in pathologic scar tissue was significantly higher than in normal and wounded skin. These results suggest a similar role for ACE participation in cutaneous pathologic scar formation as in the cardiovascular system.^27^ Tang *et al.* showed that angiotensin II induces mRNA and protein expression of type I collagen gene in human dermal fibroblasts through an TGF-β1-dependent pathway which were abolished by the angiotensin type 1 (AT1) receptor antagonist.^28^

Angiotensin II can cause upregulation and activation of TGFβ via 2 distinct signaling pathways. In the first pathway, TGF-β after binding to receptor activin receptor like kinase 5 (ALK5) which phosphorylates Smad2/3 induce recruitment of Smad4 and subsequently cause nuclear translocation of the Smad2/4 or Smad3/4. The second pathway involves regulation of mitogen activated protein kinase (MAPK) signaling including extracellular signal-regulated kinase (ERK), and p38. Moreover, Liu et al used skin fibroblasts of human hypertrophic scar and observed that angiotensin II activated Akt phosphorylation and phosphoinositide 3 kinase (PI 3-K) cascade, partially via the transactivation of epidermal growth factor (EGF) receptor, which could be blocked by an angiotensin II type 1 receptor-specific blocker.^26^

In an investigation of formation of hypertrophic scars in a rabbit ear wound model by Uzun et al, authors indicated that as decrease in angiotensin II level leads to nonselective reduction of TGF-β isomers, which is only desirable for TGF-β1 and TGF- β2 in the prevention of hypertrophic scars, addition of TGF-β3 to an ACE–inhibitor may be synergistic for scar improvement in the wound area.^29^ Ehanire investigated granulation tissue contraction, a main feature of hypertrophic scar, by angiotensin II and through angiotensin II type 1 (AT1) receptor and downstream TGF-β signaling pathway. Higher level of AT1-receptor expression was detected in scar. Fibroblast contraction and migration was induced through angiotensin II activated AT1-receptor. In-vivo experiments determined that while angiotensin II stimulated wound contraction, absence of AT1-receptor diminished granulation tissue contraction.^30^

Ren *et al.* investigation of collagen metabolism regulation by angiotensin II in skin tissues from diabetic mice model indicated that in primary cultured fibroblasts prompted collagen synthesis, mediated through modulating matrix metalloproteinase-1 (MMP-1)/tissue inhibitor of metalloproteinase-1 (TIMP-1) balance, was accompanied by elevated TGF-β expression which could be inhibited by losartan, an angiotensin II receptor blocker. These findings present evidence for a TGF-β-dependent mechanism in angiotensin II induced changes in skin fibrosis.^31^ It was demonstrated that after bleomycin induced lung injury, concentration of lung angiotensin II increases and precedes the increment in lung collagen. Administration of ramipril (an ACE inhibitor) and losartan (an angiotensin II type 1 receptor antagonist) attenuated TGF-beta expression, and collagen deposition. These observations not only suggest the important role of ANG II in the fibrotic response to acute injury, at least mediated in part via TGF-beta, but also manifest the potential of ACE inhibitors, as widely used clinical drugs, for therapy of fibrotic diseases.^32^

Safaee Ardekani *et al.* studied the effect of topical captopril, an inhibitor of angiotensin II production, against hypertrophic scar formation in New Zealand white rabbits and showed that the scar elevation index was significantly lower in the captopril treated wounds compared to the vehicle treated wounds. On the other hand, an increase in collagen organization was achieved by the captopril treated wounds.^33^ To our knowledge the present study shows in a randomized, double-blind design that topical enalapril significantly decreases the clinical parameters of hypertrophic scar and also itching as an indirect indicative of scar improvement. Furthermore, enalapril proved to be clinically safe for patients with low incidence of adverse drug reactions and acceptable cost effectiveness. Consequently, more studies are needed to assess the optimal effective concentration as well as the frequency of application and also to verify the efficacy of enalapril in large populations as a suitable alternative in the treatment of hypertrophic scars. Other ACE inhibitors stand to be investigated to assess whether these compounds can be added to the anti-hypertrophic scar armamentarium.

## CONFLICT OF INTEREST

The authors declare no conflict of interest.
